# Evaluation of the agreement by examiners according to classifications of third molars

**DOI:** 10.4317/medoral.17483

**Published:** 2011-12-06

**Authors:** Carla J. Lima, Luiz C. F. Silva, Marcelo R. S. Melo, Jadson A. S. S. Santos, Thiago S. Santos

**Affiliations:** 1 Undergraduate student, Faculty of Dentistry, Federal University of Sergipe, Aracaju, Brazil; 2 Adjunct Professor of Oral and Maxillofacial Surgery, Faculty of Dentistry, Federal University of Sergipe, Brazil; 3 Master’s student in Health Sciences Program, Faculty of Dentistry, Federal University of Sergipe, Brazil; 4 Doctoral’s student in Oral and Maxillofacial Surgery and Traumatology, University of São Paulo, Ribeirão Preto, Brazil

## Abstract

Objectives. This study recorded and evaluated the intra- and inter-group agreement degree by different examiners for the classification of lower third molars according to both the Winter’s and Pell & Gregory’s systems. Study Design. An observational and cross-sectional study was realized with forty lower third molars analyzed from twenty digital panoramic radiographs. Four examiner groups (undergraduates, maxillofacial surgeons, oral radiologists and clinical dentists) from Aracaju, Sergipe, Brazil, classified them in relation to angulation, class and position. The variance test (ANOVA) was applied in the examiner findings with significance level of p<0.05 and confidence intervals of 95%. Results. Intra- and inter-group agreement was observed in Winter’s classification system among all examiners. Pell & Gregory’s classification system showed an average intra-group agreement and a statistical significant difference to position variable in inter-group analysis with greater disagreement to the clinical dentists group (p<0.05). Conclusions. High reproducibility was associated to Winter’s classification, whereas the system proposed by Pell & Gregory did not demonstrate appropriate levels of reliability.

** Key words:** Agreement, reproducibility, classifications, third molars.

## Introduction

The third molars frequently present in a wide range of anatomic positions and angulations which commonly result in a high degree of dental impaction. Whether it be for prophylactic, orthodontic and prosthetic reasons or for the diagnosis of several associated pathologies, the surgical removal of these teeth is one the most performed dentoalveolar procedures in oral and maxillofacial surgery (1).

Traditionally, both the Winter’s and Pell & Gregory’s systems propose to classify the inclinations and positions of the third molars based on the relation among the dental longitudinal axis, occlusal plane and ascending mandibular ramus (2). Radiological individual anatomy, demographic aspects and operative factors are considered important variables to the determination of surgical difficulties and postoperative complication risks (3).

These methods have been extensively adopted and applied in clinical practice and also in several types of studies which have employed a range of study design, from systematic reviews to correlational prospective analysis with pre-, trans- and postopera-tive variables (4-6). However, there has been no previous research in the literature which has proved the reproducibility of such classifications nor the objectivity of their utilization as clinical and scientific parameters (7).

Therefore, the current study aims: 1) to register the angulation, class and position of lower third molars by four groups of examin-ers through analysis of digital panoramic radiographs and; 2) to estimate and evaluate the agreement degree intra- and inter-group of examiners, in order to investigate the reliability of both the Winter’s and Pell & Gregory’s classifications.

## Materials and Methods 

This observational and cross-sectional study design was developed in a private dental clinic from Aracaju, Sergipe, Brazil over the course of 12 months and was approved by the Ethics Committee in Research of University Hospital, Federal University of Ser-gipe, under number of protocol 0068.0.107.000-09. Initially, fifty digital panoramic radiographs which presented at least one lower third molar with indication for surgical removal were involved in this research. 

From this initial sample, the study included patient radiographs of both males and females aged from 18 to 30 years. On the other hand, it excludes those radiographs which presented inappropriate technical standard, absence or bad positioning of the isolateral second molar and molars largely destroyed or reduced to roots fragments. Subsequently, twenty of the initial fifty radiographs were selected for inclusion in the research, totalizing 40 teeth for the agreement analysis.

These digital radiographs were impressed in photographic paper (Fujicolor Crystal Archive 20x28 cm), numbered from 01 to 20 and organized systematically. They were then individually evaluated by sixty examiners equally divided into two groups: under-graduates and professionals. Only undergraduates who had already completed the discipline of Oral and Maxillofacial Surgery were included in the group of 30 students. In turn, the 30 professionals were composed by 10 oral and maxillofacial surgeons (OMFS), 10 oral radiology specialists and 10 clinical dentists from Aracaju/Sergipe. 

The lower third molars were analyzed and the examiners findings recorded on an objective formulary according to the following variables: angulation, class and position. Information about the criteria defined by both the Winter and the Pell & Gregory systems for third molars classification ( Table 1) were provided to all examiners before the radiographic analysis which ensured that this study was not evaluating the individual knowledge of examiners but rather to estimate the agreement degree for either of the two classification systems. During this study both the buccal and lingual inclinations of the Winter’s system were disregarded since an occlusal radiography, which would be required to record them, was not available. 

All statistical analysis was done with the SPSS (version 17.0) statistical package. The level of significance was p< 0.05 and data were presented with 95% confidence intervals for the mean where applicable. Differences from baseline relative frequencies were equality distributed by Levene test and then the Variance Test – ANOVA was applied for the four groups of examiners, in order to analyze if there was significant difference inter-groups. Only in the statistically significant variables, a post-test (Bonferroni Test) was used with the purpose of identifying which examiner group demonstrated disagreement. The agreement intra-group of examiners was evaluated by descriptive analysis.

## Results

For Winter’s classification, from 40 lower third molars involved in this study, the mesioangular was the predominant angulation for all groups (30.25 ± 0.96). The most frequent class and position for Pell & Gregory’s classification was II (28.5 ± 2.38) and B (21.25 ± 1.5), respectively.

A high intra-group agreement exceeding 78% was observed in all groups for Winter’s classification. Pell & Gregory’s classification presented an average intra-group agreement with means below 76%. ( Table 2) provides percentage means of in-tra-group agreement to angulation, class and position variables.

**Figure 1:**
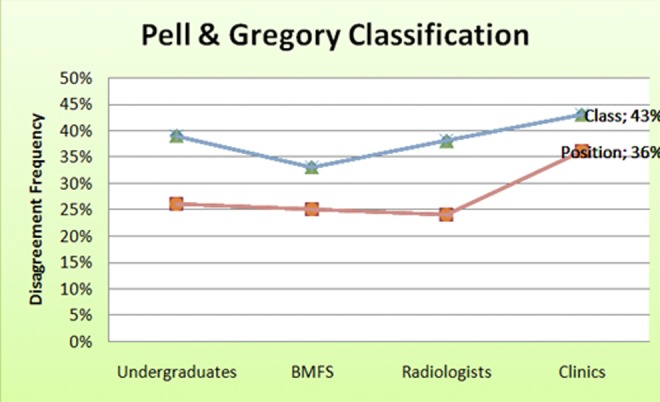
Intra-group disagreement analysis.

The intra-group disagreement for Pell & Gregory’s classification was similar among undergraduates, OMFS and radiologists. However, the clinical dentists group showed great disagreement means, either to class or position variables (Fig. 1).

**Table 1:**
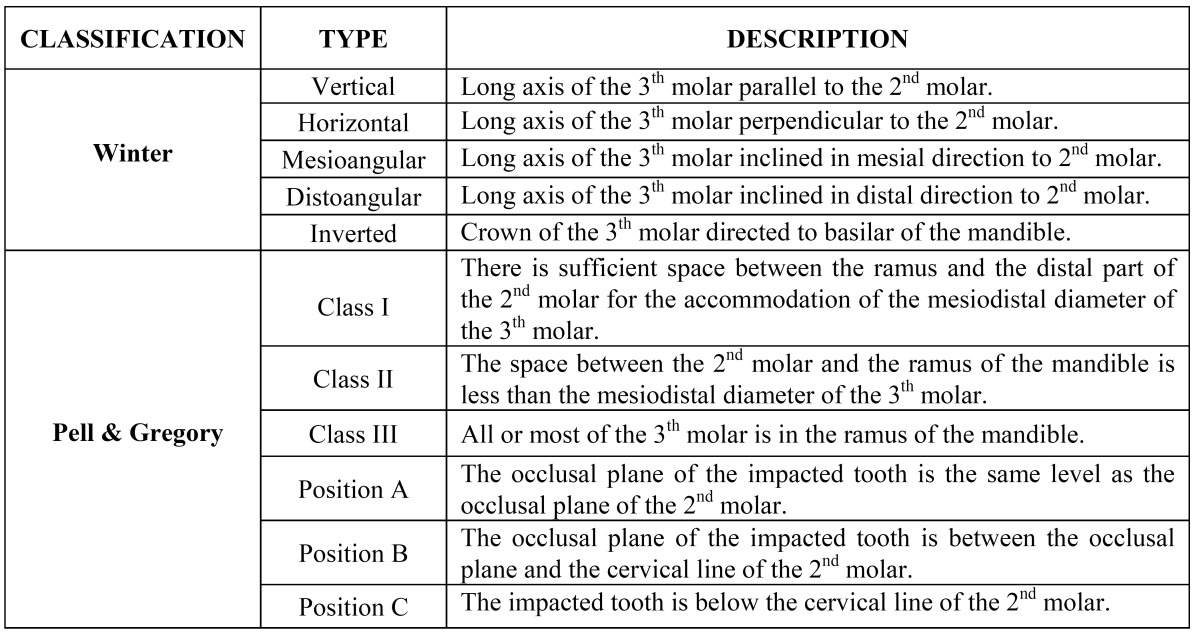
Winter’s and Pell & Gregory’s criteria (2).

**Table 2:**
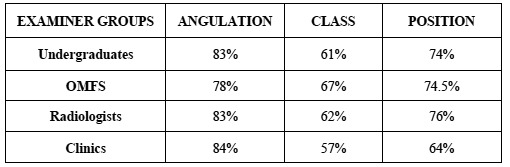
Intra-group agreement according to the classifications (mean).

Regarding the inter-group agreement among the four examiner groups, the Winter’s classification system did not show differences in the means (p=0.58). No statistical significant difference was also found to class variable for Pell & Gregory’s clas-sification (p=0.11), however a value of p<0.05 was identified to position variable, demonstrating agreement absence. 

**Figure 2:**
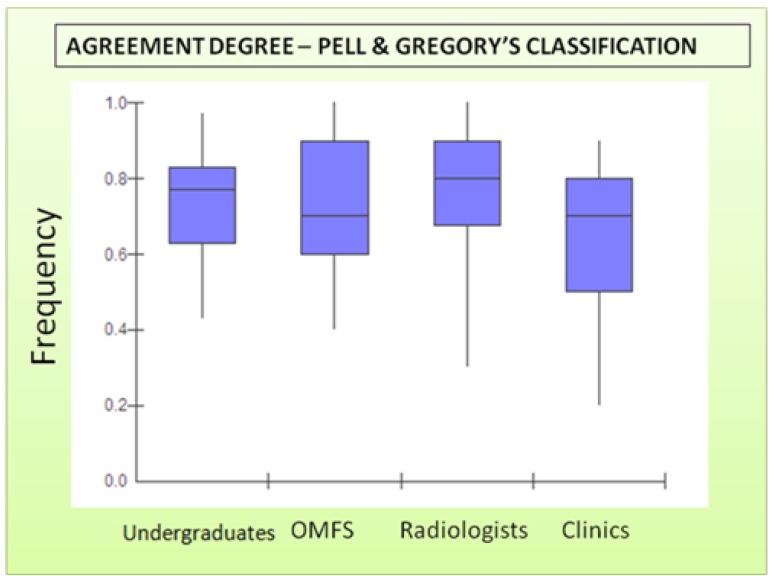
Boxplot to inter-group agreement for position variable. The top and bottom of the Box are the 25th and 75th percentiles. The line drawn through the middle of the Box in the median (the 50th percentile). The length of the Box is the interquartile range (IQR). The Box represents the middle 50% of the data. Median = 0.77, 0.70, 0.80, 0.70 for undergraduates. OMFS, radiologists and clinics, respectively. The interquartile range for clinics Box shows an enlargement with less frequent responses for the agreement.

**Figure 3:**
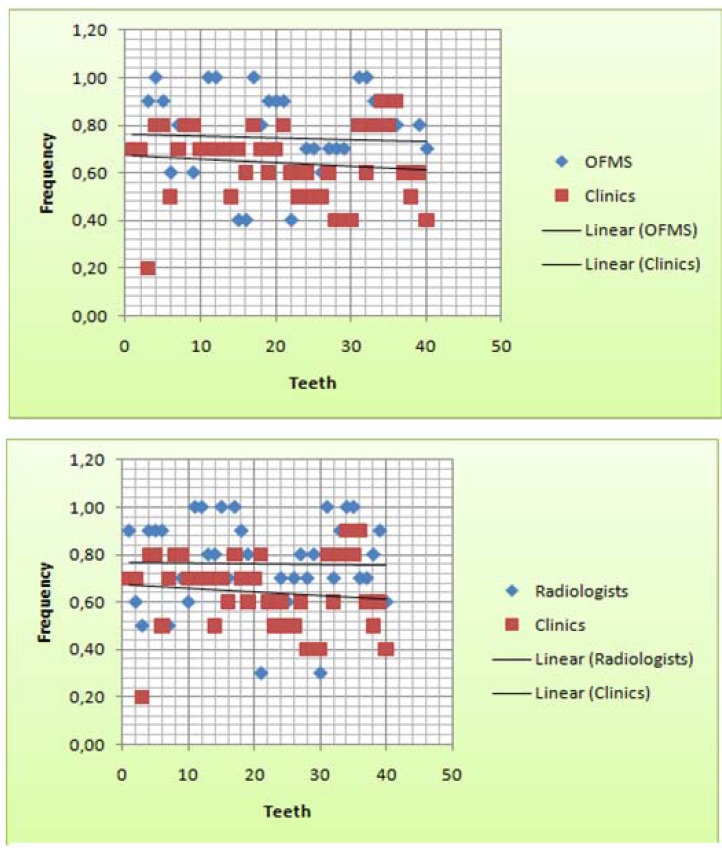
Scatter plot to dispersion of professional inter-group agreement for position variable. Scatter plot showed a greater dispersion (since 85% frequency to 20%) for the clinics group and in a global evaluated the others professional groups were mixed in the same area.

In multiple comparisons of the examiner groups on a 2x2 basis to position variable (Pell & Gregory’s classification), the clinical dentists was the only group that showed statistically significant differences (p<0.05) which indicates lower agreement by the examiners in inter-group analysis. (Figs. 2, 3) illustrate the inter-group distribution in relation to position variable, highlighting the clinical dentists’ disagreement. 

## Discussion

In clinical practice, the knowledge about the quality of the diagnostic methods is essential. Therefore, new diagnostic scales or classification systems must undergo significant review and analysis which would demonstrate satisfactory levels of measurement reliability and validity (8). Reliability can be defined as a repetition or reproduction of results obtained under a similar methodol-ogy, whereas validity, expressed by tests of sensitivity and specificity, determines whether the research truly measures which it was intended or how truthful the research results are (9).

Therefore classifications for impacted third molars should be based on reliable and valid clinic-radiological parameters which provide greater accuracy in

patient assessment and treatment planning. Nevertheless, the existent systems for classifying the third molars have widely been accepted without prior scientific evaluation, mainly in comparative researches (10).

Bui et al. (11) positively correlate the mesioangular impactions with a higher risk for operative and inflammatory complications. Yuasa et al. (12) found that depth and ramus relationship/space available were associated with severe pain and facial swelling. Regarding the surgical difficulty, Pederson’s index was developed from both the Winter’s and Pell & Gregory’s criteria (13). Similarly, Yuasa et al. (14) associate a third molar with depth degree C, Class III, bulbous roots or a combination of these three factors on panoramic images to a complicated extraction, although they regard that this classification is not totally valid for assessing surgical difficulty.

This study was conducted to evaluate intra- and inter-group reproducibility of both the Winter’s and Pell & Gregory’s classification systems for 40 lower third molars. The results obtained from Winter’s system show the high reliability of this system, either by intra- or inter-group analysis. The small disagreement degree observed may be related to the few categories that compose this classification as reported by Almendros-Marqués et al. (10).

In turn, Pell & Gregory’s classification demonstrated an average agreement degree for intra-group analysis, while a position disagreement was observed in inter-group examination. These results may reflect the greater chance of induced error in presence of a larger number of possible combinations between classes and positions. Perhaps this doubt may be expressed in the high frequency of intermediate impaction categories as class II and position B which may have been easily confounded with categories immediately before or after them.

Chaves Yuasa et al. (12) suggest that a high degree of dental inclusion (Class III/Position C) requires a surgical technique more invasive, while García et al. (15) regard Pell & Gregory’s scale as inconsistent predictor of surgical difficulty in the extraction of vertical impacted lower third molars. The results of this study approximates those of García et al. in that they did not show high levels of the reliability and validity to this classification.

The similar agreement among the undergraduates and the other groups (OMFS and radiologists) may indicate the didactic characteristic of this system and its clinical limitation, mainly by the difficulty of classifying non-vertical molars as it is related in literature (15,16). 

As this study depended greatly on the examiner performance and classification criteria, the disagreement of a single group (clini-cal dentists) for Pell & Gregory’s system may be more related to the difficulty of the examiners to use the classifica-tion system appropriately rather than to the classification system itself. It suggests that further research should be conducted to validate the existing classifications or to define new methods which induce smaller superposed categories.
